# Economic evaluation of disease elimination: An extension to the net-benefit framework and application to human African trypanosomiasis

**DOI:** 10.1073/pnas.2026797118

**Published:** 2021-12-09

**Authors:** Marina Antillon, Ching-I Huang, Kat S. Rock, Fabrizio Tediosi

**Affiliations:** ^a^Epidemiology and Public Health, Swiss Tropical and Public Health Institute, 4123 Allschwil, Switzerland;; ^b^University of Basel, 4001 Basel, Switzerland;; ^c^Zeeman Institute, University of Warwick, Coventry CV4 7AL, United Kingdom;; ^d^Mathematics Institute, University of Warwick, Coventry CV4 7AL, United Kingdom

**Keywords:** eradication, elimination, economic evaluation, mathematical modeling

## Abstract

The global health community has earmarked a number of diseases for elimination or eradication, and these goals have often been praised on the premise of long-run cost savings. However, decision makers must contend with a multitude of demands on health budgets in the short or medium term, and costs per case often rise as the burden of a disease falls, rendering such efforts beyond the cost-effective use of scarce resources. In addition, these decisions must be made in the presence of substantial uncertainty regarding the feasibility and costs of elimination or eradication efforts. Therefore, analytical frameworks are necessary to consider the additional effort for reaching global goals, like elimination or eradication, that are beyond the cost-effective use of country resources. We propose a modification to the net-benefit framework to consider the implications of switching from an optimal strategy, in terms of cost-per-burden averted, to a strategy with a higher likelihood of meeting the global target of elimination or eradication. We illustrate the properties of our framework by considering the economic case of efforts to eliminate the transmission of *gambiense* human African trypanosomiasis (gHAT), a vector-borne, parasitic disease in West and Central Africa, by 2030.

## Significance

While the health economic implications of disease elimination have been discussed before, the combination of uncertainty, cost effectiveness, and elimination has not been tackled before. We propose a modification to the net-benefit framework to explicitly consider the implications of switching from an optimal strategy, in terms of cost-per-burden averted, to a strategy with a higher likelihood of meeting the global target of elimination. The modification proposed yields a methodology to quantify the efficiency of elimination and to aid discussions among stakeholders with different objectives. We apply our method to strategies against human African trypanosomiasis in three settings, but this method is flexible enough that it can be applied directly to any simulation-based studies of disease elimination efforts.

The successful eradication campaigns of smallpox and rinderpest have curried political support for the elimination or eradication of transmission (EEOT) of other diseases. Thus far, regional elimination has been achieved for land-transmitted rabies in Europe and for malaria in large parts of the world. Yet, due to the strong correlation between many of the diseases targeted for EEOT and poor sanitation, health infrastructure, or overall material conditions, such disease campaigns are disproportionately focused in low-resource settings, bringing to the fore important questions about the efficiency of EEOT efforts (1, 2).

On the one hand, expensive EEOT interventions are often justified on the basis of future cessation of activities; one front loads the expenses on a disease on the premise that public health activities can cease in the not-too-distant future, at which time investments will be recovered. Smallpox eradication is claimed to have saved, within just a few years, billions of dollars (3). However, falling just short of EEOT could be the worst of all possible scenarios: one has diverted increasing resources from other purposes, but one cannot cease activities and recover investments. In part because of the risk of failure and the accelerating per-case cost of campaigns near the end game, such efforts might not be considered an efficient use of resources from the perspective of decision makers with limited budgets contending with a variety of health challenges. For instance, there were 22 to 176 wild-type poliomyelitis cases reported yearly between 2016 and 2020 but eradication campaigns cost approximately $1 billion annually (4, 5). Guinea worm disease (GWD) caused only 54 reported human cases in 2019, yet it is the subject of campaigns that cost approximately $30 million annually (6, 7). The eradication targets of both diseases have experienced delays of 23 and 10 y, respectively, therefore stalling the promise to recover investments (7, 8). It is for these reasons that EEOT campaigns have occasionally been called into question (8, 9). The funding for these efforts comes in part from global health stakeholders with large portfolios (e.g., the Bill and Melinda Gates Foundation, the Global Fund, the World Health Organization) working alongside country-level ministries of health, and therefore resources dedicated to the “last mile” of EEOT could potentially be diverted to cost-effective programs targeting other diseases.

To explore, scrutinize, and provide insights into questions surrounding EEOT, the epidemiology and health economics fields have a rich toolbox that captures the nonlinear transmission dynamics, temporal features, and economic implications of disease “control”–the disease reduction that occurs without necessarily achieving EEOT. Separately, a few studies have tried to grapple with questions around the economic implications of EEOT by employing game-theoretic approaches (3, 10, 11). These studies, usually focused on vaccine-preventable diseases, have designated different levels of coverage (e.g., vaccine coverage) necessary for control and EEOT, and the difference in costs between control and EEOT strategies constitutes the price for elimination (3, 10, 11). While making the problem theoretically tractable, applications of such methods are quite narrow: in practice, addressing the burden of a disease may require various distinct activities, and parsing the activities that contribute uniquely to control versus EEOT is not always possible, as each activity contributes to both objectives to varying degrees.

Moreover, there is a lot of uncertainty in whether strategies lead to EEOT. In practice, one cannot purchase EEOT with certainty, as GWD and polio have shown; one merely invests in activities that are conducive to EEOT, and therefore, the absence of probabilistic thinking in previous literature fails to capture a key component of the decision-making process. Frameworks with multiple objectives are exceedingly rare, and while some work has been done to consider policy analyses of stakeholders with differing fundamental objectives within a modeling framework [i.e., Probert et al. (12)], such approaches have seldom been developed for cost-effectiveness analyses (13) and never for analyses taking into account EEOT objectives.

Here we develop a framework that can handle 1) strategies that have different probabilities of EEOT, 2) where activities are not easily classified as exclusively control or “elimination” activities, and 3) where multiple objectives–specifically disease burden reduction and EEOT–are transparently considered. We extend the net-benefit framework, useful for decision analysis in the presence of uncertainty, to evaluate the cost effectiveness of public health strategies while explicitly outlining the “premium” of elimination or the additional resources that are necessary to bring a country’s activities in line with global goals. One important feature of our framework is that it is operationalized within a Monte Carlo simulation framework, which makes it a simple extension to ubiquitous approaches for decision analysis in the face of uncertainty, thereby being applicable to a wide array of problems.

We then apply our framework to *gambiense* human African trypanosomiasis (gHAT) in three distinct regions of the Democratic Republic of Congo (DRC). The three regions highlight the strengths of our framework and its applications under different circumstances: circumstances of certainty, uncertainty, and where more than two strategies could potentially interrupt disease transmission.

## Background

Our objective is to introduce an extension to the net-benefit framework to account for the resource implications of aligning potentially incongruous objectives of efficiency and elimination of transmission (EOT).

The keystone metric of value for money in cost-effectiveness analysis is the incremental cost-effectiveness ratio (ICER), which we touch on briefly as a point of departure for the net-benefit framework. The ICER is defined as the ratio of the difference in costs, ΔC, and the difference in health effects, ΔE of two interventions,

$$\text{ICER}=\frac{\Delta C}{\Delta E},$$

where the change in costs and health effects is computed as the net difference in costs and effects between strategies. Effects are usually denominated in disability-adjusted life years (DALYs), a metric that is comparable across diseases. For the purpose of our analysis, the effects will be distinguished between DALYs and the probability of EOT.

Between two strategies, a second strategy will be considered cost effective compared to the first one (the comparator strategy) if the ICER does not exceed a health planner’s willingness to pay ($\lambda^{\;\text{WTP}}$) per DALY averted (14, 15):

$$\text{ICER}=\frac{\Delta C}{\Delta \text{DALYs}}\leq \lambda^{\text{WTP}}.$$

The health planner’s $\lambda^{\;\text{WTP}}$ per DALY averted is equivalent to the ICER of the least efficient strategy (the strategy with the highest ICER) in the portfolio (16, 17). Recently the World Health Organization (WHO) has advocated that cost-effectiveness results should be shown at a range of WTP values; Table 1 contextualizes select WTP values for DRC, the country that is the subject of our examples.

**Table 1.** Contextualizing willingness-to-pay values $\left(\lambda_{\;\text{DALY}}^{\;\text{WTP}}\right)$ for low-income settings

$\lambda_{\text{DALY}}^{\text{WTP}}$ $	Rationale
0	This is cost saving or cost neutral over the chosen time horizon of the analysis. It should be noted that annual expenditure or budgets are not necessarily static across the whole period for all (or any) strategies.
250	Two studies that modeled the real investments made across countries estimated that the investments in DRC are $5 to $230 per DALY averted in 2013 US dollars (18) or $54 to $69 per DALY averted in 2015 US dollars (19). We rounded up to $250 for convenience.
500	Approximately equivalent to the annual per capita gross domestic product (GDP) of DRC in 2018, which was the definition of a “very cost-effective” strategy as delineated in the WHO CHOICE program (20).
1,500	Approximately equivalent to three times the annual per capita GDP of DRC, which was the definition of a “cost-effective” strategy as delineated in the WHO CHOICE program (20).

Not surprisingly, uncertainty in costs and DALYs exists, in particular with regard to diseases in populations that are difficult to study or diseases that have been historically neglected. There exists literature devoted to the difficulties of accounting for parameter uncertainty when calculating ICERs, arising from the fact that ICERs are ratios, while remaining consistent with the economic principles on which cost effectiveness is grounded (14, 21, 22). The net-benefit framework was therefore developed to circumvent some of the issues surrounding ICERs and uncertainty. The net-monetary benefit (NMB) is a simple arithmetic rearrangement of the ICER into a linear additive formulation:

$$\begin{array}{l} \frac{\Delta C}{\Delta \text{DALYs}}\leq \lambda^{\text{WTP}}\\ \Delta C\leq \lambda^{\text{WTP}}\times \Delta \text{DALYs}\\ 0\leq \lambda^{\text{WTP}}\times \Delta \text{DALYs}-\Delta C\\ 0\leq \text{NMB}(\lambda^{\text{WTP}}), \end{array}$$

where $\lambda^{\;\text{WTP}}$ is the monetary value for DALYs avoided. The linear additive formulation circumvents the technical issues with samples of ratios (22). Given a Monte Carlo sample of *N* iterates of the disease and cost model, a strategy is preferred over the comparator if the expected NMB exceeds zero:

$$0\leq E(\text{NMB}(\theta_{i},\lambda^{\text{WTP}})),$$

where *θ* is the parameter vector and i∈{1,…,N} are the iterates of each parameter. In a multiple-strategy decision analysis between *J* strategies, the preferred strategy is the strategy that maximizes $E(\text{NMB}(\theta_{i},\lambda^{\text{WTP}}))$:

$$\underset{j\in 1:J}{\text{argmax}}\;E\left(\text{NMB}\left(\text{Strat }j,\theta_{i}|\lambda^{\text{WTP}}\right)\right).$$

The differences refer to the difference between the comparator strategy (*j* = 1) and any alternative strategy j∈2:J in the analysis.

Simultaneously, the framework allows for a probabilistic interpretation of cost effectiveness by conditioning on $\lambda^{\text{WTP}}$:

$$\mathbb{P}(\text{Strat}\;j\;\text{is}\;\text{CE}|\lambda^{\text{WTP}})=\frac{1}{N}\sum_{i=1}^{N}M(j,\theta_{i}),$$

where

$$M(j,\theta_{i})=\{\begin{array}{ll} 1 & \underset{j\in 1:J}{\text{argmax}}\text{ NMB}(\text{Strat }j,\theta_{i}|\lambda^{\text{WTP}})\\ 0 & \text{Otherwise} \end{array}.$$

The algorithm therefore presents a measure of certainty that the strategy with the highest expected NMB is optimal over all other strategies, given by the proportion of samples where the strategy has the highest NMB of all strategies (14, 23).

The value judgments implied here are quite simple: The only relevant values are net costs (including the benefit of cost savings for the program by averted disease) and DALYs averted. Other considerations not accounted for include the socioeconomic distribution of the beneficiaries vs. remaining disease victims, ethical considerations, etc. (24).

### Economic Evaluation Framework for Multiple Objectives

Our proposal makes explicit the relationship between the WTP of dual objectives for averting disease burden, denominated in DALYs averted, and elimination, while taking into account the uncertainty in achieving those objectives and the concomitant costs:

$$\begin{array}{l} \text{Net}\;\text{Monetary}\;\text{and}\;\text{Elimination}\;\text{Benefits}\;(\text{NMEB}(\theta_{i}))\\ =\;100\lambda_{\text{EOT}}^{\text{WTP}}\times \Delta I_{\text{EOT}}(\theta_{i})+\lambda_{\text{DALY}}^{\text{WTP}}\times \Delta \;\text{DALYs}(\theta_{i})-\Delta \text{C}(\theta_{i}), \end{array}$$

where the indicator function, $\Delta I_{\text{EOT}}$ is 1 if EOT is achieved by only one strategy and 0 if neither strategy or both strategies achieve EOT (for parameter set *θ_i_* and strategy *j*):

$$\Delta I_{\text{EOT}}(\theta_{i})=I_{\text{EOT}}^{\text{Strat}\;\text{B}}(\theta_{i})-I_{\text{EOT}}^{\text{Strat}\;\text{A}}(\theta_{i}).$$

The use of $100\lambda_{\;\text{EOT}}^{\;\text{WTP}}\times \Delta I_{\;\text{EOT}}(\theta_{i})$ incorporates the WTP to raise the probability of elimination. While the term $\lambda_{\;\text{DALY}}^{\;\text{WTP}}$ is interpreted as the highest price paid to avert an additional DALY, $\lambda_{\;\text{EOT}}^{\;\text{WTP}}$ is interpreted as highest price paid per additional point increase in the probability of elimination.

The linear additive scale allows us to decompose the resource expenditure into two portions: the portion that is justifiable based on averted disease burden and the portion of the expenditure that is justifiable by the pursuit of elimination. While elimination and reduction in disease burden are not separable or independent, which is accounted for in the dynamic transmission model, the linear form in this formulation allows us to separate the benefits related to burden reduction, measured in DALYs, from those of elimination. This circumvents the need to calculate $C_{\;\text{DALY}}$ (the cost for reducing burden, or DALYs) and $C_{\;\text{EOT}}$ (the cost for elimination), which are rarely separable, as no activity will contribute to a reduction in one metric without impacting the other.

In a manner analogous to the traditional NMB, the strategy that ought to be implemented is indicated by:

$$\underset{j\in 1:J}{\text{argmax}}\;E(\text{NMEB}(\text{Strat }j,\theta |\lambda_{\text{DALY}}^{\text{WTP}},\lambda_{\text{EOT}}^{\text{WTP}})),$$

and the probability that a strategy is cost effective is

$$\mathbb{P}(\text{Strat}\;j\;\text{is}\;\text{CE}|\lambda_{\text{DALY}}^{\text{WTP}},\lambda_{\text{EOT}}^{\text{WTP}})=\frac{1}{N}\sum_{i=1}^{N}M(j,\theta_{i}),$$

where

$$M(j,\theta_{i})=\{\begin{array}{ll} 1 & \underset{j\in 1:J}{\text{argmax}}\text{ NMEB}(\text{Strat }j,\theta_{i}|\lambda_{\text{DALY}}^{\text{WTP}},\lambda_{\text{EOT}}^{\text{WTP}})\\ 0 & \text{Otherwise} \end{array}.$$

### Premium of Elimination

A useful metric easily calculated from the formulation of NMEB is the premium of elimination. We begin with a simple context: if we assume there are two strategies, which are to reach elimination with 0 and 100% certainty, respectively, and we suppose that the elimination strategy would not avert any additional DALYs over the nonelimination strategy (e.g., detection and treatment are superb), then the expected premium of elimination, given here by ${\text{Premium}}_{\;\text{EOT}}$, would equal the expected cost difference between the two strategies:

$$E({\text{Premium}}_{\text{EOT}}(\theta ))=\Delta E(\text{C}(\theta )).$$

However, in practice any two or more strategies are unlikely to avert the same number of DALYs; in fact, most often the elimination-prone strategy is likely to avert some additional DALYs, albeit at a potentially high cost. The health planner has a certain $\lambda_{\;\text{DALY}}^{\;\text{WTP}}$ for those additional DALYs that the elimination strategy averts, although perhaps not a WTP that would completely bridge the gap in costs between the two strategies. In such a case, the expected ${\text{Premium}}_{\;\text{EOT}}$ is

$$\begin{array}{l} E({\text{Premium}}_{\text{EOT}}(\theta ))=\\ \max\{\Delta E(\text{C}(\theta ))-\lambda_{\text{DALY}}^{\text{WTP}}\times \Delta E(\text{DALYs}(\theta )),0\}. \end{array}$$

In other words, the more the health planner is willing to pay for DALYs averted, the lower the additional premium that will be paid for elimination.

If one strategy has both a higher probability of achieving the elimination and a relatively low incremental cost, then the premium of elimination is equal to zero, as no additional resources are needed to justify elimination beyond those resources traditionally considered to be efficient (cost effective) to avert disease.

### Premium of Elimination and NMEB

The NMEB and the ${\text{Premium}}_{\;\text{EOT}}$ are linked as follows:

$$100\lambda_{\text{EOT}}^{\text{WTP}}\times \Delta I_{\text{EOT}}(\theta_{i})\geq \Delta \text{C}(\theta_{i})-\lambda_{\text{DALY}}^{\text{WTP}}\times \Delta \text{DALYs}(\theta_{i})$$

taking the expectation of both sides of the equations:

$$100\lambda_{\text{EOT}}^{\text{WTP}}\times \Delta {\text{Pr}}_{\text{EOT}}(\theta )\geq E({\text{Premium}}_{\text{EOT}}(\theta )).$$

In other words, the premium of elimination must be smaller than the product of the between-strategy differential probability of EOT and the WTP for additional certainty of EOT. For instance, if the comparator strategy (strategy A) is the preferred strategy under the traditional NMB framework, then to select strategy B on the basis of its higher probability of elimination, the product of $\lambda_{\;\text{EOT}}^{\;\text{WTP}}$ and the differential probability must be at least as large as the ${\text{Premium}}_{\;\text{EOT}}$.

### Premium of Elimination, Discount Rates, and Time Horizons

Inadequately chosen time horizons and discount rates would impact the size of the premium of elimination. While the benefits of elimination or eradication could reach infinitely into the future, one does not generally include these infinite benefits or savings for two reasons. First, the principles of time preference generally dictate that we apply a discount rate, which would make the costs and benefits decades into the future worth almost zero in present-day terms. Second, even if no discounting were to be taken into account, most analyses of interventions of nonchronic infectious diseases have time horizons under 20 y, and picking a longer time horizon for our analysis would not allow comparability across diseases in a health planner’s portfolio (20). Finally, it is recognized that lifetime rewards for current-day strategies against infectious diseases are difficult to incorporate as the state of epidemics several decades into the future can be influenced by factors that cannot be adequately predicted (25). Therefore, we caution against inadequately short time horizons or unusually high discount rates, as this could raise the premium of elimination, and, by the same token, unusually long time horizons and low discount rates would lower the premium of elimination and compromise comparability across studies.

## Results

### Health Outcomes, Costs, and Traditional ICERs

Using a joint transmission and cost model we made projections of the epidemiological impact and resource use between 2020 and 2040 of four strategies against gHAT in three locations (Table 2 provides an overview on the component interventions, while further details can be found in *Materials and Methods*). In region 1, success or failure of the 2030 EOT goal is certain, depending on the selected strategy, but in regions 2 and 3 success and failure of the EOT goal is uncertain (Table 3).

**Table 2.** Strategies for control and elimination of gHAT in a typical endemic health district

	Strategy			
Component interventions	Mean AS*	Max AS	Mean AS & VC	Max AS & VC
Mean active screening	*✓*	*✓*	*✓*	*✓*
Additional active screening		*✓*		*✓*
Passive screening	*✓*	*✓*	*✓*	*✓*
Vector control			*✓*	*✓*
Treatment of cases	*✓*	*✓*	*✓*	*✓*

Passive screening (PS): gHAT screening that occurs in local health posts of patients who present themselves with specific gHAT symptoms. Active screening (AS): The examination of individuals in their village by mobile teams who screen and confirm cases. Treatment: Detected cases (either active or passive) are referred to the district hospital for treatment according to WHO guidelines (26). Vector control (VC): Biannual deployment of tiny targets to control the population of tsetse. Our simulation assumes that the tsetse population decreases by 80% in the first year, consistent with field studies (27–29).

* Status quo strategy.

**Table 3.** Intermediate outcomes, cost effectiveness, and elimination of transmission in three example health zones

	Mean AS	Max AS	Mean AS & VC	Max AS & VC
Region 1				
Cases	477 (144, 1,081)	463 (136, 1,047)	116 (41, 235)	120 (38, 270)
Deaths	207 (41, 614)	174 (36, 499)	54 (18, 115)	49 (16, 105)
DALYs	3,939 (886, 11,007)	3,336 (779, 9,161)	1,185 (405, 2,494)	1,077 (362, 2,280)
Δ DALYs	Comparator	602 (–191, 2,221)	2,754 (339, 8,765)	2,862 (382, 8,956)
Costs (US dollars, × 1,000)	3,101 (2,153, 4,736)	4,023 (2,734, 6,308)	3,811 (2,464, 6,007)	4,284 (2,732, 6,731)
Δ Costs (US dollars, × 1,000)	Comparator	921 (451, 1,619)	709.8 (–763.9, 2,765)	1,182 (–291.7, 3,415)
Pr. EOT	0	0	100	100
Δ Pr. EOT	Comparator	0	100	100
Region 2				
Cases	23 (1, 79)	22 (0, 92)	9 (0, 41)	10 (0, 54)
Deaths	12 (1, 42)	8 (0, 28)	5 (0, 15)	4 (0, 12)
DALYs	247 (20, 803)	167 (2, 564)	106 (1, 318)	82 (1, 262)
Δ DALYs	Comparator	80 (–87, 366)	142 (–41, 551)	165 (–21, 597)
Costs (US dollars, × 1,000)	1,029 (508, 1,841)	1,407 (637, 2,652)	1,258 (636, 2,068)	1,529 (743, 2,544)
Δ Costs (US dollars, × 1,000)	Comparator	377.5 (–164.3, 1,105)	229 (–451.9, 933.8)	499.7 (–209.8, 1,335)
Pr. EOT	79	92	100	100
Δ Pr. EOT	Comparator	13	21	21
Region 3				
Cases	65 (2, 224)	64 (1, 264)	27 (1, 84)	31 (0, 122)
Deaths	32 (1, 137)	19 (0, 89)	14 (0, 54)	10 (0, 44)
DALYs	676 (23, 2,809)	414 (4, 1,885)	336 (10, 1,245)	242 (3, 1,008)
Δ DALYs	Comparator	262 (–38, 1,133)	340 (–50, 1,684)	434 (–14, 1,926)
Costs (US dollars, × 1,000)	970 (524, 1,552)	1,164 (573, 2,058)	1,622 (869, 2,793)	1,659 (882, 3,023)
Δ Costs (US dollars, × 1,000)	Comparator	193.5 (–138.4, 599.4)	651 (16, 1,613)	689 (38, 1,763)
Pr. EOT	42	54	100	100
Δ Pr. EOT	Comparator	12	58	58

Pr. EOT: probability of elimination of transmission. DALYs: disability-adjusted life-years.

If the status quo (comparator) strategy remains in place (mean active screening [Mean AS]), there will be an average of 477 cases and 207 deaths in region 1, 23 cases and 12 deaths in region 2, and 65 cases and 32 deaths in region 3. In terms of DALYs, there will be 3,939 DALYs in region 1, 247 DALYs in region 2, and 676 DALYs in region 3. Under any strategy in all settings, the burden of disease is expected to decline, but strategies with vector control (VC) are expected to expedite this decline substantially (*SI Appendix*, Fig. S3).

Costs per year show that while strategies that include VC are more costly in the short run, the investments begin to yield returns after 2028 in region 1, after 2025 in region 2, and after 2030 in region 3 (*SI Appendix*, Fig. S4). Costs are driven by AS activities and, when applicable, by VC activities, so the timing of cessation of these activities plays an important role in the ability of ambitious investments to be recovered (*SI Appendix*, Fig. S5). Nevertheless, it is not certain that these strategies will yield cost savings over a 20-y investment period, although total costs are only marginally higher compared to the comparator (Mean AS).

### Net Monetary Elimination Benefits: Where Success and Failure Are Certain

**Fig. 1.**
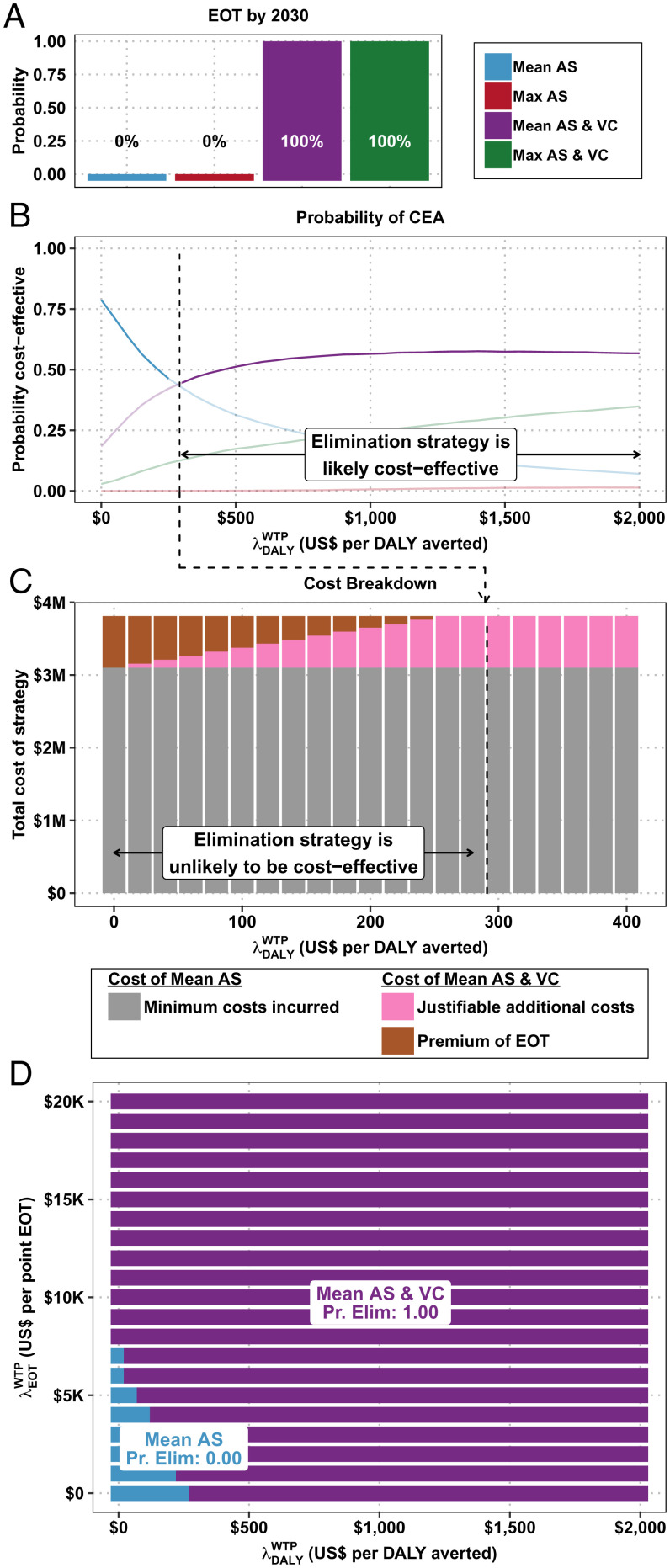
Cost-effectiveness acceptability curves (CEACs) and cost breakdown for region 1. (*A*) The probability of EOT by 2030 for each strategy. (*B*) The traditional CEACs and CEAFs. (*C*) The total cost for a strategy that reaches elimination with 100% probability and the breakdown between minimum cost strategy and justifiably additional costs and premium of elimination across $\lambda_{\text{DALY}}^{\text{WTP}}$ values. (*D*) CEAHs.

The probability of EOT in region 1 is shown in Fig. 1*A* and the results of our decision analysis under the traditional net benefits framework are shown in Fig. 1*B*. After taking into account parameter uncertainty, our analysis shows that Mean AS has an 80% probability of having the minimum cost (optimal at $\lambda_{\;\text{DALY}}^{\;\text{WTP}}$ = 0). However, if $\lambda_{\;\text{DALY}}^{\;\text{WTP}}$ > $258, the strategy Mean AS & VC is optimal with 47 to 55% probability.

In region 1, the Mean AS strategy has a 0% probability of EOT by 2030, whereas the Mean AS & VC strategy has 100% probability of EOT. The expected ${\text{Premium}}_{\;\text{EOT}}$ is shown in Fig. 1*C*. In a policy environment of low $\lambda_{\;\text{DALY}}^{\;\text{WTP}}$, any health planner must be able to justify the entire $709,800 cost difference between Mean AS and Mean AS & VC on the basis of EOT alone. When DALYs are not valued monetarily ($\lambda_{\;\text{DALY}}^{\;\text{WTP}}$ = $0), the ${\text{Premium}}_{\;\text{EOT}}$ is simply equivalent to the difference in costs between the two strategies. If, for instance, $\lambda_{\;\text{DALY}}^{\;\text{WTP}}$ = $100, a health planner must be able to justify only a ${\text{Premium}}_{\;\text{EOT}}$ of $434,400, as the other $275,400 that would be justifiable by DALYs averted. In a policy environment of a $\lambda_{\;\text{DALY}}^{\;\text{WTP}}$ = $300, the strategy that reaches elimination is entirely justifiable on the health gains achieved (DALYs averted), and the ${\text{Premium}}_{\;\text{EOT}}$ is therefore $0.

Fig. 1*D* shows the optimal choice of strategy for a range of $\lambda_{\;\text{DALY}}^{\;\text{WTP}}$ and $\lambda_{\;\text{EOT}}^{\;\text{WTP}}$ values. In a policy environment where $\lambda_{\;\text{DALY}}^{\;\text{WTP}}$ = 0 and $\lambda_{\;\text{EOT}}^{\;\text{WTP}}$ = 7,098 per additional probability point of EOT, the optimal strategy guarantees elimination, as that is the $\lambda_{\;\text{EOT}}^{\;\text{WTP}}$ that justifies the $709,800 premium of elimination.

### Net Monetary Elimination Benefits: Where Success and Failure Are Uncertain

Region 2 illustrates a setting where the comparator strategy (Mean AS) has a 79% probability of EOT, and therefore the binary conception of control or elimination strategies fails to adequately capture the decision maker’s dilemma.

**Fig. 2.**
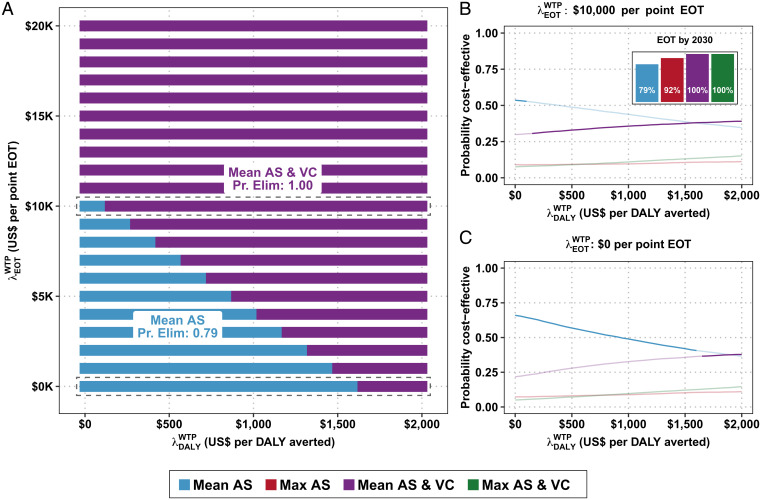
(*A*) CEAHs for region 2. Along the *x* axis is the cost-effectiveness threshold for averting disease burden, $\lambda_{\;\text{DALY}}^{\;\text{WTP}}$, and along the *y* axis is the cost-effectiveness threshold for EOT, $\lambda_{\;\text{EOT}}^{\;\text{WTP}}$, to raise the probability of EOT by 2030 by one percentage point. (*B* and *C*) CEACs and CEAFs. *B*, *Inset* is the probability of each strategy achieving EOT by 2030.

The conditions for which an economically optimal strategy maximizes the probability of EOT are shown in Fig. 2. If $\lambda_{\;\text{DALY}}^{\;\text{WTP}}$ = 0, a strategy that almost ensures EOT–Mean AS & VC–is optimal if $\lambda_{\;\text{EOT}}^{\;\text{WTP}}$ > $10,684 per additional percentage point, and the ${\text{Premium}}_{\;\text{EOT}}$ is $229,000 (*SI Appendix*, Table S3). If, however, $\lambda_{\;\text{DALY}}^{\;\text{WTP}}$ = $500, then the ${\text{Premium}}_{\;\text{EOT}}$ is $158,000 and the $\lambda_{\;\text{EOT}}^{\;\text{WTP}}$ > $7,377. At $\lambda_{\;\text{DALY}}^{\;\text{WTP}}$ = 1,500, then ${\text{Premium}}_{\;\text{EOT}}$ decreases to $16,000 and $\lambda_{\;\text{EOT}}^{\;\text{WTP}}$ = $764.

**Fig. 3.**
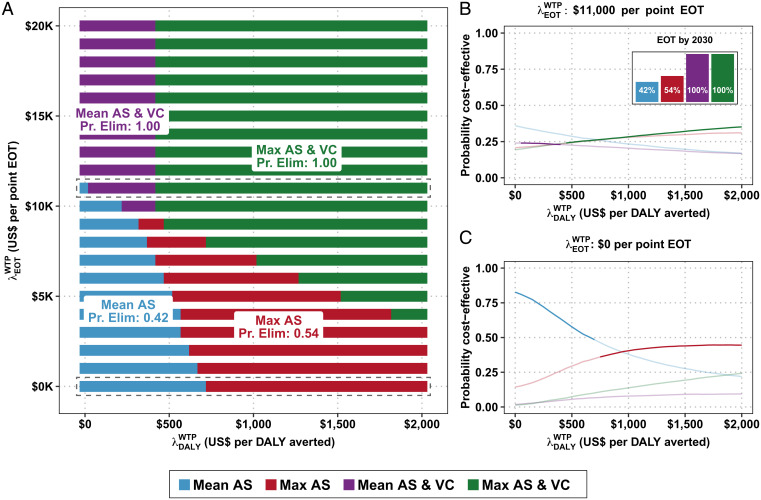
(*A*) CEAHs for region 3. Along the *x* axis is the cost-effectiveness threshold for averting disease burden, $\lambda_{\;\text{DALY}}^{\;\text{WTP}}$, and along the *y* axis is the cost-effectiveness threshold for EOT, $\lambda_{\;\text{EOT}}^{\;\text{WTP}}$, to raise the probability of EOT by 2030 by one percentage point. (*B* and *C*) Traditional CEAFs. *B*, *Inset* is the probability of each strategy achieving EOT by 2030.

Our most complex setting, region 3, is shown in Fig. 3. Under the traditional net-benefit framework, either the Mean AS or the maximum (Max) AS strategy is cost effective at $\lambda_{\;\text{DALY}}^{\;\text{WTP}}$ values consistent with historical investment levels in low-income countries (Fig. 3*C*), but these strategies have only a 42 and 54% probability of EOT, respectively (Fig. 3*B*). Without an investment in elimination justified by benefits that extend beyond the averted DALYs, achievement of EOT is uncertain.

**Fig. 4.**
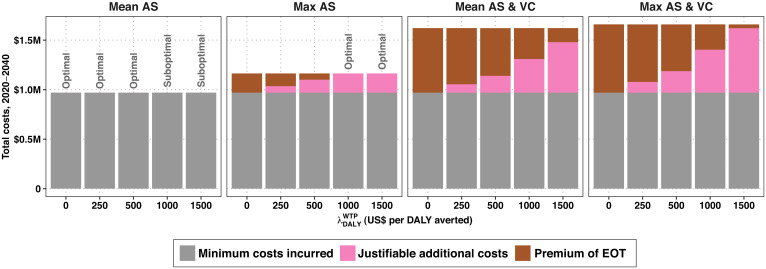
Premium of elimination in region 3, across different values of $\lambda_{\text{DALY}}^{\text{WTP}}$, contextualized in Table 1.

At $\lambda_{\;\text{DALY}}^{\;\text{WTP}}$ = 0, then a decision maker must have a $\lambda_{\;\text{EOT}}^{\;\text{WTP}}\geq$ $15,747 and incur a ${\text{Premium}}_{\;\text{EOT}}$ = $194,000 to bolster the chances of elimination from 42 to 54% or $\lambda_{\;\text{EOT}}^{\;\text{WTP}}\geq$ $11,210 and incur a ${\text{Premium}}_{\;\text{EOT}}$ = $651,000 to bolster the probability of elimination from 42 to >99%. Therefore, as long as one does not value DALYs averted, the switch from Mean AS to Max AS would incur a lower premium, but switching to Mean AS & VC would be more efficient on the basis of per-point probability of reaching EOT (Fig. 4 and *SI Appendix*, Table S4).

If, however, the policy environment is one where $\lambda_{\;\text{DALY}}^{\;\text{WTP}}$ = $500, then the Mean AS & VC strategy has become dominated by both objectives: Max AS will avert more DALYs than Mean AS & VC because it treats more extant infections in the short term, and Max AS & VC is more efficient at both averting DALYs and raising the probability of EOT on a per-point basis. To maximize the probability of EOT when $\lambda_{\;\text{DALY}}^{\;\text{WTP}}$ = $500, one would deploy Max AS & VC, which has a ${\text{Premium}}_{\;\text{EOT}}$ = $472,000, equivalent to $\lambda_{\;\text{EOT}}^{\;\text{WTP}}$ > $8,934. At $\lambda_{\;\text{DALY}}^{\;\text{WTP}}$ = $1,500, the strategy Max AS is cost effective, so the ${\text{Premium}}_{\;\text{EOT}}$ of Max AS & VC is $237,000 and $\lambda_{\;\text{EOT}}^{\;\text{WTP}}$ > $5,172.

## Discussion

Much of the early literature about cost effectiveness and decision making in the presence of uncertainty was about analytical methods to do what is now easily executed through simulation (15, 21, 30, 31). Although simulation of a wide array of epidemiological and policy questions is ubiquitous, the interpretation of those simulations must rest within frameworks that decompose uncertainty and frame economic optimization in a manner that is in line with economic theory. The existing tools in the health economics toolbox generally answer the question, “are public health efforts economically justified?” but when the global health community has set ambitious goals, such as elimination of infectious diseases, questions of resource use across different administrative levels (local, national, regional, and global) do not fit neatly into existing frameworks and one asks, “to what degree are public health objectives economically justified by various objectives?” In the context of EEOT of infectious diseases, there may be a tension between EEOT objectives and maintaining an efficient use of scarce resources, as defined by dollars per DALYs averted (32). This is particularly salient because the same resources–in-kind, capital, and financial–that vie for EEOT efforts could be diverted to address other urgent health goals.

We have presented an extension to the net-benefit framework to inform decisions that contain an elimination objective that may stand at odds with concerns about efficient resource allocation. Our proposed framework explicitly models the additional premium for elimination activities in the presence of uncertainty.

The illustrative analysis shows that in region 1, EOT is nearly impossible with the comparator strategy, but EOT is cost effective at a relatively low $\lambda_{\;\text{DALY}}^{\;\text{WTP}}$ > $300, therefore yielding $\lambda_{\;\text{EOT}}^{\;\text{WTP}}$ = $0 (Fig. 1 and *SI Appendix*, Table S2). The other regions present more complicated scenarios: EOT is likely in region 2 (79%) and moderately likely in region 3 (42%) even with the comparator strategy (Mean AS), but the value for money in terms of EOT objectives varies by location. In region 2 when $\lambda_{\;\text{DALY}}^{\;\text{WTP}}$ = $0, the ${\text{Premium}}_{\;\text{EOT}}$ = $229,000 and on a per-percentage-point basis $\lambda_{\;\text{EOT}}^{\;\text{WTP}}$ = $10,684 (*SI Appendix*, Table S3). Region 3 has a moderate ${\text{Premium}}_{\;\text{EOT}}$ of $651,000 for switching to a strategy sure to deliver EOT, but on a per-percentage-point basis, the cost ($\lambda_{\;\text{EOT}}^{\;\text{WTP}}$) is $11,210 (*SI Appendix*, Table S4). By contrast, although the ${\text{Premium}}_{\;\text{EOT}}$ is highest in region 1 ($709,800), it is the place where investments justified on the ground of elimination alone are the most efficient, $\lambda_{\;\text{EOT}}^{\;\text{WTP}}$ = $7,098. In short, while one could justify strategies that maximize the probability of elimination with a sufficiently high $\lambda_{\;\text{DALY}}^{\;\text{WTP}}$ in regions 1 and 2, that would not be possible in region 3. In this way, if the current framework is employed simultaneously for various settings, as we have done here, or for multiple diseases marked for elimination, the results could aid in allocating resources to those locations or diseases where efforts for EOT are most efficient, starting where $\lambda_{\;\text{EOT}}^{\;\text{WTP}}$ is lowest.

While we contend that it is often the case that objectives of public health interventions may span beyond those defined by DALYs, it is important that stakeholders define the benefits borne by elimination above and beyond the reduction of DALYs, as the reduction of DALYs already goes a long way in conferring many of the benefits sought after: reduction of disease risk to neighboring regions, reduction of stigma, etc. (9). Still, elimination may provide benefits that low prevalence does not: an absolute certainty that disease risk is equal among all social strata (33), the reduction to zero of risks of future epidemics and other unknowable risks, etc. In discussions among stakeholders, the benefits of burden reduction versus elimination must be outlined in a manner specific to the context of the disease–a task that goes beyond the methodological preoccupations of cost-effectiveness analyses as presented here.

Existing literature on the economic evaluation of potential elimination strategies has been limited to diseases of person-to-person transmission, often considering one modality of prevention so that control and elimination are reduced to a matter of the degree of coverage of vaccination (as with smallpox) or of treatment (as with HIV). This has made the analyses parsimonious, but it is unclear how such approaches generalize to real-world scenarios, where there are multiple modalities of prevention (3, 10, 11). In the case of gHAT as well as multiple other diseases, it is not clear that one activity alone is the key to elimination. Our framework therefore expands the categories of diseases that could be analyzed via a common set of metrics amenable to simulation analyses: $\lambda_{\;\text{DALY}}^{\;\text{WTP}},\;\lambda_{\;\text{EOT}}^{\;\text{WTP}}$, and ${\text{Premium}}_{\;\text{EOT}}$. Alternatively, one could employ the same framework for another goal (i.e., reduction in inequality) to which there are benefits not captured by DALYs averted.

Our contention is that EOT is often not something that can be purchased outright, but rather something that can be invested in, and therefore the willingness to pay must be expressed in terms of the increased probability of reaching the goal. However, ascertaining the value of $\lambda_{\;\text{EOT}}^{\;\text{WTP}}$ would probably be easier by asking stakeholders how much they would pay for elimination in a context where the probability of EOT is certain such that the probability is 0 or 1 with different strategies (like in region 1).

One alternative measure of the opportunity costs of elimination or eradication can be expressed in terms of the DALYs incurred when we forgo an efficient program (in terms of cost per DALYs averted) in favor of a less efficient program likely to achieve elimination of a disease. In a perfectly efficient health portfolio, one would displace a program that has a cost-effectiveness ratio equal to the $\lambda_{\;\text{DALY}}^{\;\text{WTP}}$, and therefore one could calculate this opportunity cost by dividing the ${\text{Premium}}_{\;\text{EOT}}$ by $\lambda_{\;\text{DALY}}^{\;\text{WTP}}$: if a ${\text{Premium}}_{\;\text{EOT}}$ is $1 million and the $\lambda_{\;\text{DALY}}^{\;\text{WTP}}$ = $1,000, then the opportunity cost of elimination is 1,000 DALYs incurred by another disease. However, the interpretation of such an opportunity cost must be taken with caution, as the benefits for programs are not necessarily divisible if only a fraction of the program can be funded–a concern of cost-effectiveness analyses in general.

The EEOT of neglected tropical diseases is believed to drive toward the achievement of several sustainable development goals (2, 34) as well as equity, as the elimination or eradication of a disease provides the same protection against that disease to individuals across the socioeconomic spectrum (33). Under the present framework, we contend that not only is the investment on EEOT beyond its disease burden aspect (in terms of DALYs) possible within campaigns that succeed, but also these benefits are achieved in relation to how close a strategy comes to elimination in terms of its probability of success. However, one does not necessarily need to enumerate each benefit of elimination and its relation to other social values, one needs only to have reasonable evidence of the presence of stakeholders that value EEOT to justify employing the approach we have presented here. Because the data to enumerate additional nonhealth benefits of elimination could be missing or weak, we propose an approach that circumvents such challenges by calculating the lower bound for the necessary willingness to pay for elimination.

### Limitations

The total costs of EEOT will inevitably be affected by the size of the population that must be treated, which involves the concepts of critical community size, the degree of connectivity of metapopulations, and importation probability—all factors that we do not examine here. Some diseases are worth eliminating in small patches because even one imported case will not reestablish transmission, while other diseases are only worth eliminating if elimination can be achieved throughout large interconnected networks of settlements (35–37).

Unlike many neglected tropical diseases, gHAT interventions have been very heterogeneous, even across the same administrative district, and so two regions with the same number of reported cases in 2017 may have quite different underlying epidemiology. We captured this uncertainty by utilizing posterior parameters from various regions; however, the present results are not designed to be representative of a single area. Tailored models, fitted to longitudinal case and intervention data, will yield more reliable location-specific recommendations for gHAT strategies.

It is worth noting that the premium of elimination is not a subsidy, although this number could be used to inform a subsidy. Incurring outlays in the short term may require financing products (i.e., loans, grants) even in the context of a strategy that could be cost saving in the long run.

While we do not address issues surrounding the elimination of diseases and their inequitable distribution, there is a budding literature regarding such concerns termed “equity-enhanced cost-effectiveness analysis (CEA),” “extended CEA,” and “distributional CEA” (32, 38–40).

### Conclusions

With this method, one can evaluate whether health-resource efficiency, in terms of DALYs, is enough to justify efforts that bring about elimination. The difference between the preferred strategy to reach each objective and the joint preferred strategy may set the basis for discussions on joint actions between stakeholders. This framework makes tractable a plethora of analyses that could inform elimination priorities even when the benefits of elimination cannot be enumerated exactly.

## Materials and Methods

### An Application: Human African Trypanosomiasis

gHAT is a parasitic infection caused by *Trypanosoma brucei gambiense* and transmitted by tsetse (biting flies). gHAT infections are almost always fatal if untreated, and at the peak of the epidemic in the late 1990s, it is suspected that up to tens of thousands of cases went undetected and untreated (41, 42). In 2012, the WHO marked gHAT for elimination of transmission by 2030 (43). While gHAT has historically burdened 14 countries, the DRC remains the most affected, accounting for over 74% of the worldwide caseload (44).

Here we employed a previously published model of gHAT transmission fitted to historic data from three health zones in DRC: Kwamouth, in Mai Ndombe province; Mosango, in Kwilu province; and Sia, in Kwilu province. Details about these health zones are in *SI Appendix*, section 1 and in a previous publication (45). Previously published models are based on epidemiological data provided by the WHO Human African Trypanosomiasis (HAT) Atlas (46). We selected these locations as they provide interesting illustrative examples of the NMEB framework.

### Health Effects, Costs, and Cost Effectiveness

All modeling choices are described in previous publications (45, 47, 48) and summarized in *SI Appendix*, section 1. The model provided projections of future case reporting as well as unobservable features such as transmission events, disease burden, and unreported deaths under alternative strategies for each year between 2020 and 2040 (47). The four strategies made up of a combination of interventions are shown in Table 2 and illustrated in *SI Appendix*, Fig. S1.

We then applied a model of the resource use for these strategies (48) to estimate the costs and health burden accrued and averted in terms of cases, deaths, and DALYs. Costs were denominated in 2018 US dollars. Both costs and health effects were discounted at a rate of 3% in accordance with standard practice (20) and we performed our main analysis from the perspective of the healthcare providers collectively over a 20-y time horizon (2020 to 2040).

Uncertainty was accounted for in two ways: 1) Uncertainty in all model parameters was propagated via Monte Carlo simulation, drawing 10,000 random samples from probability distributions chosen to characterize the extant uncertainty in each parameter in accordance with established practice (23), and 2) the model-simulated stochasticity in case detections.

Because we are concerned with cost effectiveness and uncertainty, we construct cost-effectiveness acceptability frontiers (CEAFs), which denote the optimal strategy (in terms of cost effectiveness) at a range of willingness-to-pay values (15). Finally, we develop the cost-effectiveness acceptability heatmaps (CEAHs), a form of two-way CEAF with both *λ* values as *x* and *y* axes and the preferred intervention indicated by the color of the area of the heatmap. We use no predefined thresholds for WTP values, as we aim to provide guidance rather than prescription.

## Supplementary Material

Supplementary File

## Data Availability

R code and simulation results data have been deposited in Open Science Framework (https://OSF.IO/FH6CA) (49).
