# Supplementary material for: Economic evaluation of disease elimination: An extension to the net-benefit framework and application to human African trypanosomiasis
Source: Proc Natl Acad Sci U S A. 2021 Dec 9;118(50):e2026797118. doi: 10.1073/pnas.2026797118 (PMC8685684; doi:10.1073/pnas.2026797118)
Supplement: Supplementary File [file pnas.2026797118.sapp.pdf]

Supplementary Information for

Economic evaluation of disease elimination: an extension to the net benefits framework and application to human African trypanosomiasis

Marina Antillón, Ching-I Huang, Kat S Rock, Fabrizio Tediosi

Corresponding author: Marina Antillón.
E-mail: marina.antillon@swisstph.ch

This PDF file includes:

Supplementary text
Figs. S1 to S5
Tables S1 to S4
SI References

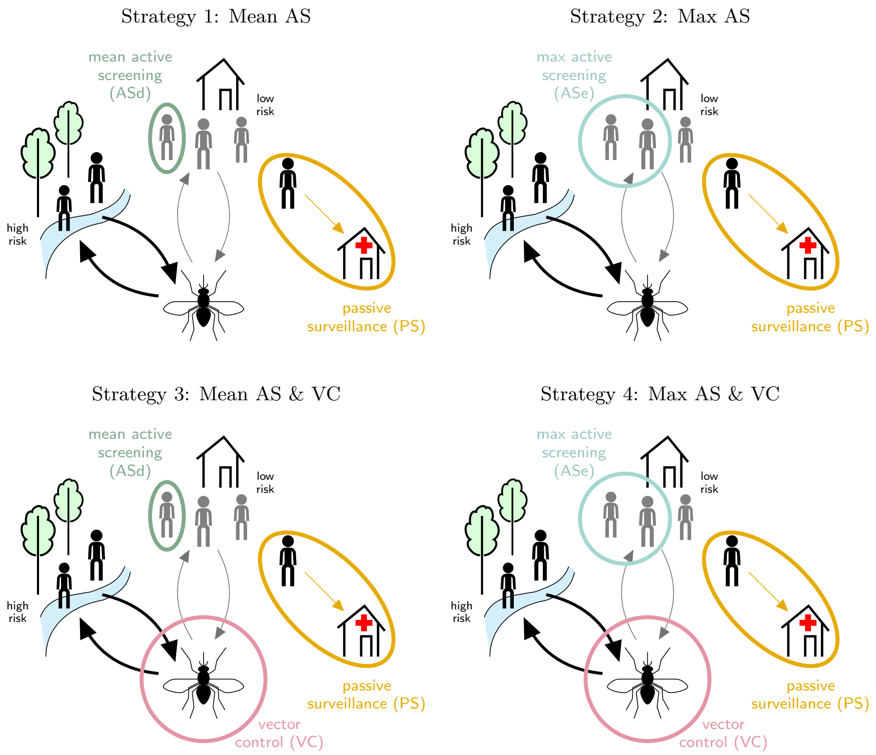

Fig. S1. Model of strategies against gHAT in DRC including active screening (AS) by mobile teams, passive surveillance (PS) in fixed health facilities. Cases detected by either mode are treated. In two strategies ('Mean AS' and 'Mean AS & VC') the active screening coverage is equal to the mean number screened during 2014–2018. In two other strategies ('Max AS' and 'Max AS & VC'), the coverage is the maximum number screened during 2000–2018. In strategies 3 and 4 vector control (VC) is simulated assuming a 80% tsetse density reduction after one year. PS is in place under all strategies. Reprinted from ref. 3, which is licensed under CC BY 4.0 (3).

Supporting Information Text

1. Supplementary methods

A. Disease model. We employed a previously published dynamic, deterministic transmission Susceptible-Exposed-Infected-Recovered-Suceptible (SEIRS) model. While the model is detailed elsewhere (1, 2), briefly, we simulated gHAT illness and vector transmission in a compartmental model described by deterministic features, simulated using a set of ordinary differential equations. The stochastic nature of the observations was conferred by sampling from the infected prevalence to simulate those individuals who are diagnosed and report for treatment, either in a fixed health facility or to a mobile screening unit, and whether imperfect diagnostics correctly detect cases or identifies non-infected people as cases.

A Markov Chain Monte Carlo (MCMC) approach was previously used to generate posterior parameter sets for which the model outputs match the longitudinal data for different regions of the Democratic Republic of Congo (DRC) during 2000–2016. In the present study we selected three example regions – Kwamouth (Region 1), Mosango (Region 2), and Sia (Region 3) – of the 168 originally fitted in order to highlight diverse results of our new framework. The locations used in the current analysis are described in Table S1.

Table S1. Descriptive summaries of three health zones.

Characteristic	Kwamouth	Mosango	Sia
Province	Mai-Ndombe	Kwilu	Kwilu
Population (2016 est.)	131,022	125,076	114,041
Area (km^2)	14,589	2,606	2,990
Active screening as a percent of 2016 population (mean; max)	48; 69	34; 60	19; 30
HAT testing centers (2014 est.)	5	1	1
Yearly incidence per 10,000 (2014–2018)	8.70	0.99	1.02
WHO Incidence category (2014–2018)	Moderate	Low	Moderate
Vector control extent (linear km)	432	210	210
Vector control density (targets per linear km)	40	40	40

N.B.: For Kwamouth, the extent of riverbank where vector control must be performed is informed by planned activities. For Mosango and Sia, assumptions regarding vector control are based on the experience in places of similar size.

B. Strategies and intervention model.

B.1. Medical Interventions. While the projections of strategies have been detailed in previously published manuscripts (3, 4), we provide a brief sketch of the features of the simulation within the context of the current analysis. To determine the number of cases detected by screening as well as the time lived with disease for cases that were never detected, we simulated a diagnostic algorithm combined with the prevalence determined by the transmission model. Although diagnostic algorithms are elaborate in practice, we simulated a simple algorithm that would capture the major features of the real process (5, 6). Suspects in traditional active or passive surveillance activities are first screened by the Card Agglutination tests for Trypanosomiasis (CATT) or rapid diagnostic tests (RDTs). Serologically-positive suspects then have blood drawn for microscopy, and if trypanosoma are found, a patient undergoes a lumbar puncture to stage their disease (early infection is "stage 1" and late is "stage 2") and determine care. For those ineligible for oral fexinidazole treatment, staging of the disease is done via lumbar puncture and followed by either treatment with pentamidine (stage 1) or nifurtimox-eflornithine combination treatment (NECT, stage 2) (3, 7).

B.2. Vector control. To control the population of tsetse, special "Tiny Targets" have been developed that stand on riversides – typical tsetse habitat – and deliver deadly insecticide upon contact (8–11). The advantage of this method of control of disease is that it breaks the chain of transmission, even when some cases cannot be reached for treatment. Activities entail placing Tiny Targets alongside the riverbanks twice per year for as many years as it takes to see a decline in the transmission of cases. The impact of these activities on tsetse density has been documented elsewhere (10, 11) and its impact on disease transmission has been evaluated in one modeling study for Chad (12) and through analysis of case reporting in Guinea (13).

In our study, we calculate that vector control will have to be deployed along 437 km of riverbank in Kwamouth but only 210 km of riverbank in Mosango and Sia. The reason is that we assumed that smaller health zones like Mosango and Sia would need vector control activities closer to those in Yasa Bonga, a health zone of 2,606 km^2 (11). Kwamouth, by contrast, spans 14,589 km^2 and contains two hot spots of transmission and therefore requires a broader treatment of riverbanks (3).

B.3. Interventions in the endgame. In simulating the end-game, we also assumed that additional confirmatory procedures such as video microscopy (or lab-based tests) are being used to elevate the previous high specificity of screening algorithms ($\approx$ 99.9%) to 100% in this context of diminishing prevalence (2). We further simulate the impact of stopping active screening and vector control interventions (where applicable) based on observing three consecutive years of zero cases reporting (in either active screening or passive surveillance). Our algorithm would allow restarting active screening should further cases later arise through continuing passive detection. This cessation criterion is not only plausible in practice (it is unlikely that interventions would continue indefinitely) but also is important in capturing the impact of stopping transmission and therefore saving future intervention costs.

C. Key outputs. The key outputs of the dynamic and diagnostic models include mortality in undetected cases, detected cases in stages 1 and 2, and DALYs before and after presenting to care for all interventions. The number of people actively screened, and number (if any) of vector deployments performed each year is recorded.

D. Elimination of Transmission. Elimination of transmission (EOT) is assumed when the underlying transmission (not detected cases) falls below 1 new infection per year (this proxy threshold is necessary when using a deterministic model to approximate peri-elimination dynamics and has been used elsewhere (4, 14). The metric of interest in this paper is regional EOT (where baseline activities must remain to prevent re-establishment) rather than eradication because we are not treating the issue of importation of cases (15). This is also related to the World Health Organization's gHAT goal for 2030 which is global EOT to humans (16).

E. Health impact. We measured health impact by combining the epidemiological outputs from the dynamic/screening model with a probability tree that simulates the branching process of treatment progression and disease outcomes.

In accordance with the WHO interim guidelines on gHAT treatment (7), we assumed that treatment would consist of 1800 mg of fexinidazole for four days and 1200 mg of fexinidazole for six days for most patients in stage 1 and 2 disease. We assumed that patients would be treated in equal parts on an inpatient and outpatient basis (as directly-observed therapy). Patients who were either under 6 years old or under 20 kg in weight, undergo a lumbar puncture to determine disease stage and are sorted accordingly into pentamidine or NECT inpatient treatment.

We simulated the disease process separately for stage 1 and stage 2 disease, and a small proportion of stage 1 cases are assumed not cooperate with care or to undergo treatment failure, and are thus added to the number of cases that undergo stage 2 care.

The disease and treatment probability tree model is deterministic, formalized mathematically through the product of conditional probabilities of the outcome at each stage of disease and treatment progression (Fig S2). The disease tree model for stage 1 includes: follow-up (for patients lost-to-follow-up), the presence of side effects, treatment success or failure, diagnosis (in the case of treatment failure), and progression to stage 2 treatment (if applicable). For stage 2, additional steps include death due to treatment and the process of rescue treatment (for patients who fail first-choice treatment for stage 2).

Health burden is denominated in DALYs, but we report cases and deaths for the benefit of the reader (Table 3). The probability of EOT is denominated as a probability, and we treat it separately to DALYs averted.

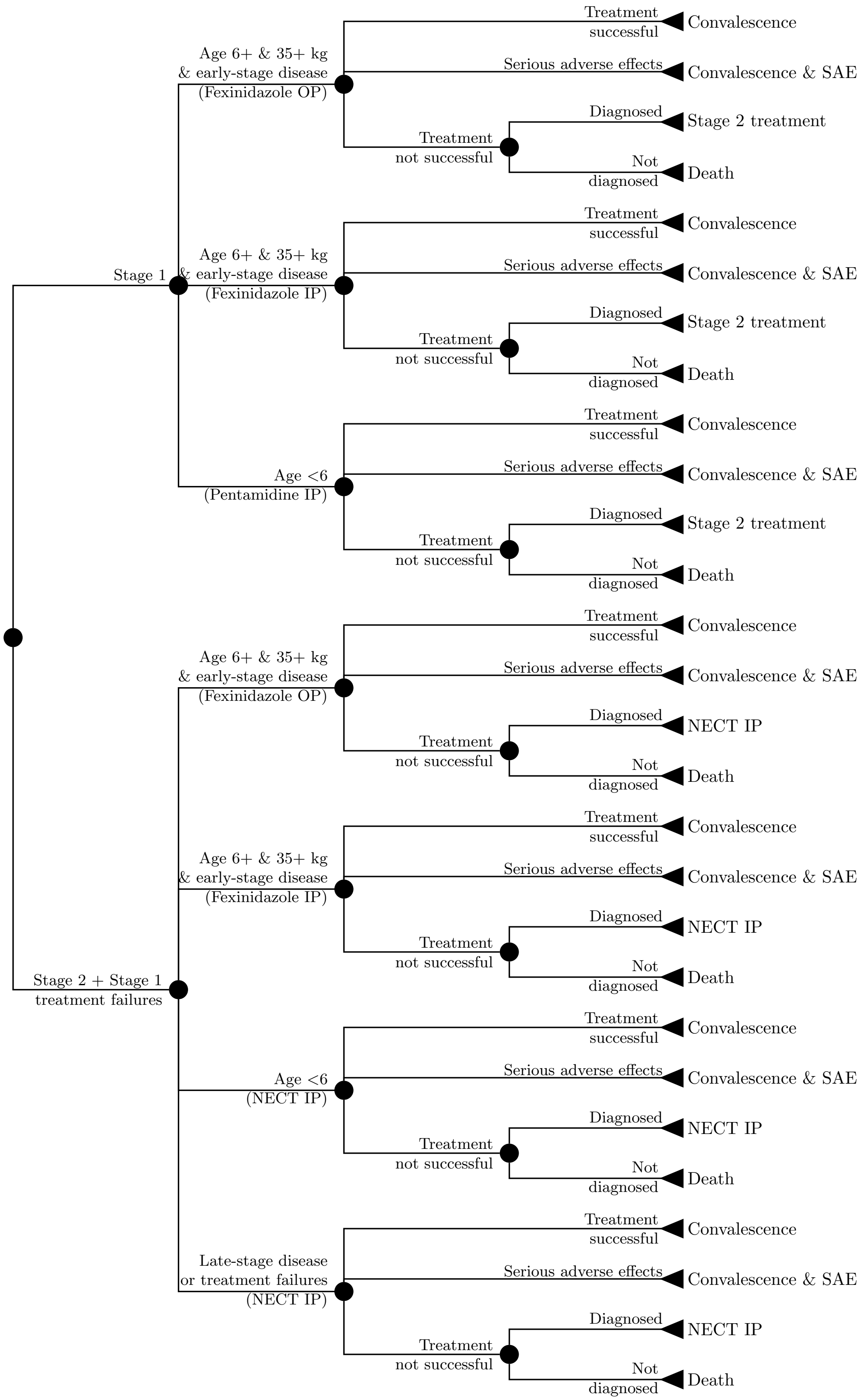

Fig. S2. Model of treatment for gHAT stages 1 and 2. Figure reproduced under the creative commons licence from Antillon $et\ al$ (3).

F. Costs. We performed our analysis using the health payer perspective, and therefore, only direct medical costs are included in our analysis. The cost function structure is identical to that described in (3), and therefore we only briefly describe the main features here, but assumptions behind consumption vary slightly.

Intervention costs are estimated as the product of unit costs and appropriate units as informed by the extant literature on interventions and our collaborators in DRC. Accelerating costs per case found towards the end-game is the results from a decreasing number of cases.

Disease costs are linked to the probability tree model of treatment progression, and include diagnosis, confirmation, and staging, as well as the cost of the drug itself and the administration. Costs values are drawn from the literature. Where no cost data existed, WHO CHOICE costs were used. All costs are inflated to 2018 US$ values.

As with the health impact, we compute costs using a 20-year time-horizon to allow time to consider the economic rewards of elimination by 2030. However, any other choice of time horizon would be equally amenable to use in this new framework.

G. Cost-effectiveness.

G.1. Risk-averse vs risk-neutral decision-makers. Due to an interesting feature in probabilistic simulations, there is a possibility that the optimal cost-effective strategy is not the strategy with the highest probability of cost-effectiveness. One might note that in situations in which the probability distributions of the parameters in the simulation are asymmetric and have long tails, some strategies might have a lower median than a mean, thus yielding low probabilities of cost-effectiveness among all simulations but a high expected value of the NMB. A risk-neutral decision-maker would look only to maximize the health returns on a given WTP, and thus would choose the strategy with the highest expected NMB:

$$\underset{k \in 1:J}{\operatorname{argmax}}\, \mathbb{E}(\mathrm{NMB}(k, \theta_i | \lambda^{\mathrm{WTP}}))$$

A risk-averse decision-maker, on the other hand, might consider both the expected net benefit and the probability of being cost-effective: the decision-maker may select the strategy that has the highest chance of being cost-effective, or perhaps the decision-maker may select the strategy with a higher expected net benefit if the incremental risk is not too large.

This debate is beyond the scope of the paper, and we will proceed with an assumption of a risk-neutral decision-maker.

G.2. Time horizons. We chose a 20-year time-horizon starting in 2020 to allow the economic rewards of elimination by 2030 to be reaped.

2. Supplemental results

Table S2. Intermediate outcomes, cost-effectiveness, and efficiency of elimination in Region 1.

	Mean AS	Max AS	Mean AS & VC	Max AS & VC
**Basic outputs**				
Cases	477 (144, 1,081)	463 (136, 1,047)	116 (41, 235)	120 (38, 270)
Deaths	207 (41, 614)	174 (36, 499)	54 (18, 115)	49 (16, 105)
DALYs	3,939 (886, 11,007)	3,336 (779, 9,161)	1,185 (405, 2,494)	1,077 (362, 2,280)
ΔDALYs	Comparator	602 (-191, 2,221)	2,754 (339, 8,765)	2,862 (382, 8,956)
Costs (USD, × 1000)	3,101 (2,153, 4,736)	4,023 (2,734, 6,308)	3,811 (2,464, 6,007)	4,284 (2,732, 6,731)
ΔCosts (USD, × 1000)	Comparator	921 (451, 1,619)	709.8 (-763.9, 2,765)	1,182 (-291.7, 3,415)
ICER	Minimum cost	Dominated	258	4,373
Pr. EOT	0	0	100	100
ΔPr. EOT	Comparator	0	100	100
**λ_{DALY}^{WTP} = 0 USD**				
NMB (USD, × 1000)	0 (0, 0)	-921.2 (-1,619, -450.7)	-709.8 (-2,765, 763.9)	-1,182 (-3,415, 291.7)
Justifiable costs (USD, × 1000)	Preferred	0 (0, 0)	0 (0, 0)	0 (0, 0)
Premium$_{EOT}$ (USD, × 1000)	Preferred	921 (451, 1,619)	710 (0, 2,765)	1,182 (0, 3,415)
ΔPremium$_{EOT}$/ΔPr. EOT (vs Preferred, USD)	Preferred	No advantage	7,098	11,821
ΔPremium$_{EOT}$/ΔPr. EOT (incremental, USD)	Preferred	Dominated	7,098	Dominated
**λ_{DALY}^{WTP} = 250 USD**				
NMB (USD, × 1000)	0 (0, 0)	-770.6 (-1,501, -211.2)	-21.37 (-2,486, 2,287)	-466.7 (-3,096, 1,838)
Justifiable costs (USD, × 1000)	Preferred	150.6 (-47.86, 555.2)	688 (85, 2,191)	715 (95, 2,239)
Premium$_{EOT}$ (USD, × 1000)	Preferred	771 (211, 1,501)	21 (0, 2,486)	467 (0, 3,096)
ΔPremium$_{EOT}$/ΔPr. EOT (vs Preferred, USD)	Preferred	No advantage	214	4,667
ΔPremium$_{EOT}$/ΔPr. EOT (incremental, USD)	Preferred	Dominated	214	Dominated
**λ_{DALY}^{WTP} = 500 USD**				
NMB (USD, × 1000)	0 (0, 0)	-620 (-1,430, 243.9)	667 (-2,248, 4,273)	248.7 (-2,845, 3,910)
Justifiable costs (USD, × 1000)	Suboptimal	Suboptimal	Preferred	54.01 (-146.8, 280)
Premium$_{EOT}$ (USD, × 1000)	Suboptimal	Suboptimal	Preferred	418 (0, 1,839)
ΔPremium$_{EOT}$/ΔPr. EOT (vs Preferred, USD)	Suboptimal	Suboptimal	Preferred	No advantage
ΔPremium$_{EOT}$/ΔPr. EOT (incremental, USD)	Suboptimal	Suboptimal	Preferred	Dominated
**λ_{DALY}^{WTP} = 1000 USD**				
NMB (USD, × 1000)	0 (0, 0)	-318.9 (-1,379, 1,296)	2,044 (-1,813, 8,517)	1,680 (-2,418, 8,327)
Justifiable costs (USD, × 1000)	Suboptimal	Suboptimal	Preferred	108 (-293.6, 560.1)
Premium$_{EOT}$ (USD, × 1000)	Suboptimal	Suboptimal	Preferred	364 (0, 1,825)
ΔPremium$_{EOT}$/ΔPr. EOT (vs Preferred, USD)	Suboptimal	Suboptimal	Preferred	No advantage
ΔPremium$_{EOT}$/ΔPr. EOT (incremental, USD)	Suboptimal	Suboptimal	Preferred	Dominated
**λ_{DALY}^{WTP} = 1500 USD**				
NMB (USD, × 1000)	0 (0, 0)	-17.69 (-1,404, 2,406)	3,421 (-1,469, 12,859)	3,110 (-2,036, 12,765)
Justifiable costs (USD, × 1000)	Suboptimal	Suboptimal	Preferred	162 (-440.4, 840.1)
Premium$_{EOT}$ (USD, × 1000)	Suboptimal	Suboptimal	Preferred	310 (0, 1,846)
ΔPremium$_{EOT}$/ΔPr. EOT (vs Preferred, USD)	Suboptimal	Suboptimal	Preferred	No advantage
ΔPremium$_{EOT}$/ΔPr. EOT (incremental, USD)	Suboptimal	Suboptimal	Preferred	Dominated

[1] A dominated strategy is one that has a higher cost but averts fewer DALYs or has the same or lower probability of EOT than a less expensive strategy.
[2] We do not show prediction intervals for ICERs as there are a variety of issues with the mathematical properties of such constructions (17).
[3] For context on the values of λ_{DALY}^{WTP} = that we have chosen to display, see Table 2.

Table S3. Intermediate outcomes, cost-effectiveness, and efficiency of elimination in Region 2.

	Mean AS	Max AS	Mean AS & VC	Max AS & VC
**Basic outputs**				
Cases	23 (1, 79)	22 (0, 92)	9 (0, 41)	10 (0, 54)
Deaths	12 (1, 42)	8 (0, 28)	5 (0, 15)	4 (0, 12)
DALYs	247 (20, 803)	167 (2, 564)	106 (1, 318)	82 (1, 262)
ΔDALYs	Comparator	80 (-87, 366)	142 (-41, 551)	165 (-21, 597)
Costs (USD, $\times$ 1000)	1,029 (508, 1,841)	1,407 (637, 2,652)	1,258 (636, 2,068)	1,529 (743, 2,544)
ΔCosts (USD, $\times$ 1000)	Comparator	377.5 (-164.3, 1,105)	229 (-451.9, 933.8)	499.7 (-209.8, 1,335)
ICER	Minimum cost	Dominated	1,615	11,578
Pr. EOT	79	92	100	100
ΔPr. EOT	Comparator	13	21	21
**λ^{WTP}_{DALY} = 0 USD**				
NMB (USD, $\times$ 1000)	0 (0, 0)	-377.5 (-1,105, 164.3)	-229 (-933.8, 451.9)	-499.7 (-1,335, 209.8)
Justifiable costs (USD, $\times$ 1000)	Preferred	0 (0, 0)	0 (0, 0)	0 (0, 0)
$Premium_{EOT}$ (USD, $\times$ 1000)	Preferred	377 (0, 1,105)	229 (0, 934)	500 (0, 1,335)
$\Delta Premium_{EOT}/\Delta$Pr. EOT (vs Preferred, USD)	Preferred	29,126	10,684	23,318
$\Delta Premium_{EOT}/\Delta$Pr. EOT (incremental, USD)	Preferred	Dominated	10,684	Dominated
**λ^{WTP}_{DALY} = 250 USD**				
NMB (USD, $\times$ 1000)	0 (0, 0)	-357.5 (-1,073, 177)	-193.5 (-912.2, 515.7)	-458.4 (-1,300, 271.7)
Justifiable costs (USD, $\times$ 1000)	Preferred	20 (-21.73, 91.53)	35.43 (-10.22, 137.7)	41.28 (-5.223, 149.3)
$Premium_{EOT}$ (USD, $\times$ 1000)	Preferred	357 (0, 1,073)	194 (0, 912)	458 (0, 1,300)
$\Delta Premium_{EOT}/\Delta$Pr. EOT (vs Preferred, USD)	Preferred	27,582	9,031	21,391
$\Delta Premium_{EOT}/\Delta$Pr. EOT (incremental, USD)	Preferred	Dominated	9,031	Dominated
**λ^{WTP}_{DALY} = 500 USD**				
NMB (USD, $\times$ 1000)	0 (0, 0)	-337.5 (-1,048, 189.1)	-158.1 (-889.4, 593.1)	-417.1 (-1,269, 339.8)
Justifiable costs (USD, $\times$ 1000)	Preferred	40.01 (-43.47, 183.1)	70.87 (-20.45, 275.4)	82.56 (-10.45, 298.7)
$Premium_{EOT}$ (USD, $\times$ 1000)	Preferred	337 (0, 1,048)	158 (0, 889)	417 (0, 1,269)
$\Delta Premium_{EOT}/\Delta$Pr. EOT (vs Preferred, USD)	Preferred	26,039	7,377	19,465
$\Delta Premium_{EOT}/\Delta$Pr. EOT (incremental, USD)	Preferred	Dominated	7,377	Dominated
**λ^{WTP}_{DALY} = 1000 USD**				
NMB (USD, $\times$ 1000)	0 (0, 0)	-297.4 (-1,013, 235.9)	-87.23 (-854.9, 779.6)	-334.6 (-1,225, 533.6)
Justifiable costs (USD, $\times$ 1000)	Preferred	80.02 (-86.94, 366.1)	141.7 (-40.89, 550.7)	165.1 (-20.89, 597.3)
$Premium_{EOT}$ (USD, $\times$ 1000)	Preferred	297 (0, 1,013)	87 (0, 855)	335 (0, 1,225)
$\Delta Premium_{EOT}/\Delta$Pr. EOT (vs Preferred, USD)	Preferred	22,951	4,070	15,613
$\Delta Premium_{EOT}/\Delta$Pr. EOT (incremental, USD)	Preferred	Dominated	4,070	Dominated
**λ^{WTP}_{DALY} = 1500 USD**				
NMB (USD, $\times$ 1000)	0 (0, 0)	-257.4 (-977.1, 312)	-16.36 (-817.6, 1,003)	-252 (-1,186, 766.8)
Justifiable costs (USD, $\times$ 1000)	Preferred	120 (-130.4, 549.2)	212.6 (-61.34, 826)	247.7 (-31.34, 896)
$Premium_{EOT}$ (USD, $\times$ 1000)	Preferred	257 (0, 977)	16 (0, 818)	252 (0, 1,186)
$\Delta Premium_{EOT}/\Delta$Pr. EOT (vs Preferred, USD)	Preferred	19,864	764	11,760
$\Delta Premium_{EOT}/\Delta$Pr. EOT (incremental, USD)	Preferred	Dominated	764	Dominated

[1] A dominated strategy is one that has a higher cost but averts fewer DALYs or has the same or lower probability of EOT than a less expensive strategy.
[2] We do not show prediction intervals for ICERs as there are a variety of issues with the mathematical properties of such constructions (17).
[3] For context on the values of λ^{WTP}_{DALY} = that we have chosen to display, see Table 2.

Table S4. Intermediate outcomes, cost-effectiveness, and efficiency of elimination in Region 3.

	Mean AS	Max AS	Mean AS & VC	Max AS & VC
**Basic outputs**				
Cases	65 (2, 224)	64 (1, 264)	27 (1, 84)	31 (0, 122)
Deaths	32 (1, 137)	19 (0, 89)	14 (0, 54)	10 (0, 44)
DALYs	676 (23, 2,809)	414 (4, 1,885)	336 (10, 1,245)	242 (3, 1,008)
ΔDALYs	Comparator	262 (-38, 1,133)	340 (-50, 1,684)	434 (-14, 1,926)
Costs (USD, $\times$ 1000)	970 (524, 1,552)	1,164 (573, 2,058)	1,622 (869, 2,793)	1,659 (882, 3,023)
ΔCosts (USD, $\times$ 1000)	Comparator	193.5 (-138.4, 599.4)	651 (16, 1,613)	689 (38, 1,763)
ICER	Minimum cost	740	Weakly dominated	2,875
Pr. EOT	42	54	100	100
ΔPr. EOT	Comparator	12	58	58
λ^{WTP}_{DALY} **= 0 USD**				
NMB (USD, $\times$ 1000)	0 (0, 0)	-193.5 (-599.4, 138.4)	-651.4 (-1,613, -15.83)	-689 (-1,763, -37.58)
Justifiable costs (USD, $\times$ 1000)	Preferred	0 (0, 0)	0 (0, 0)	0 (0, 0)
$Premium_{EOT}$ (USD, $\times$ 1000)	Preferred	194 (0, 599)	651 (16, 1,613)	689 (38, 1,763)
$\Delta Premium_{EOT}/\Delta$Pr. EOT (vs Preferred, USD)	Preferred	15,747	11,210	11,858
$\Delta Premium_{EOT}/\Delta$Pr. EOT (incremental, USD)	Preferred	Weakly Dominated	11,210	Dominated
λ^{WTP}_{DALY} **= 250 USD**				
NMB (USD, $\times$ 1000)	0 (0, 0)	-128.1 (-505.5, 180.6)	-566.4 (-1,507, 121.2)	-580.5 (-1,602, 141.1)
Justifiable costs (USD, $\times$ 1000)	Preferred	65.4 (-9.513, 283.2)	84.99 (-12.61, 421.1)	108.5 (-3.581, 481.5)
$Premium_{EOT}$ (USD, $\times$ 1000)	Preferred	128 (0, 505)	566 (0, 1,507)	581 (0, 1,602)
$\Delta Premium_{EOT}/\Delta$Pr. EOT (vs Preferred, USD)	Preferred	10,425	9,747	9,991
$\Delta Premium_{EOT}/\Delta$Pr. EOT (incremental, USD)	Preferred	Weakly Dominated	9,747	Dominated
λ^{WTP}_{DALY} **= 500 USD**				
NMB (USD, $\times$ 1000)	0 (0, 0)	-62.72 (-449, 325.5)	-481.4 (-1,416, 383.2)	-472.1 (-1,476, 431.8)
Justifiable costs (USD, $\times$ 1000)	Preferred	130.8 (-19.03, 566.4)	170 (-25.21, 842.2)	217 (-7.162, 963.1)
$Premium_{EOT}$ (USD, $\times$ 1000)	Preferred	63 (0, 449)	481 (0, 1,416)	472 (0, 1,476)
$\Delta Premium_{EOT}/\Delta$Pr. EOT (vs Preferred, USD)	Preferred	5,103	8,285	8,123
$\Delta Premium_{EOT}/\Delta$Pr. EOT (incremental, USD)	Preferred	5,103	Dominated	8,934
λ^{WTP}_{DALY} **= 1000 USD**				
NMB (USD, $\times$ 1000)	0 (0, 0)	68.09 (-394.3, 789.6)	-311.4 (-1,304, 1,036)	-255.1 (-1,295, 1,237)
Justifiable costs (USD, $\times$ 1000)	Suboptimal	Preferred	78.35 (-149.3, 689.6)	172.4 (-62.55, 933.8)
$Premium_{EOT}$ (USD, $\times$ 1000)	Suboptimal	Preferred	380 (0, 1,307)	323 (0, 1,274)
$\Delta Premium_{EOT}/\Delta$Pr. EOT (vs Preferred, USD)	Suboptimal	Preferred	8,283	7,053
$\Delta Premium_{EOT}/\Delta$Pr. EOT (incremental, USD)	Suboptimal	Preferred	Dominated	7,053
λ^{WTP}_{DALY} **= 1500 USD**				
NMB (USD, $\times$ 1000)	0 (0, 0)	198.9 (-365.1, 1,352)	-141.5 (-1,234, 1,792)	-38.07 (-1,204, 2,134)
Justifiable costs (USD, $\times$ 1000)	Suboptimal	Preferred	117.5 (-224, 1,034)	258.5 (-93.82, 1,401)
$Premium_{EOT}$ (USD, $\times$ 1000)	Suboptimal	Preferred	340 (0, 1,305)	237 (0, 1,235)
$\Delta Premium_{EOT}/\Delta$Pr. EOT (vs Preferred, USD)	Suboptimal	Preferred	7,428	5,172
$\Delta Premium_{EOT}/\Delta$Pr. EOT (incremental, USD)	Suboptimal	Preferred	Dominated	5,172

[1] A dominated strategy is one that has a higher cost but averts fewer DALYs or has the same or lower probability of EOT than a less expensive strategy.
[2] We do not show prediction intervals for ICERs as there are a variety of issues with the mathematical properties of such constructions (17).
[3] For context on the values of λ^{WTP}_{DALY} = that we have chosen to display, see Table 2.

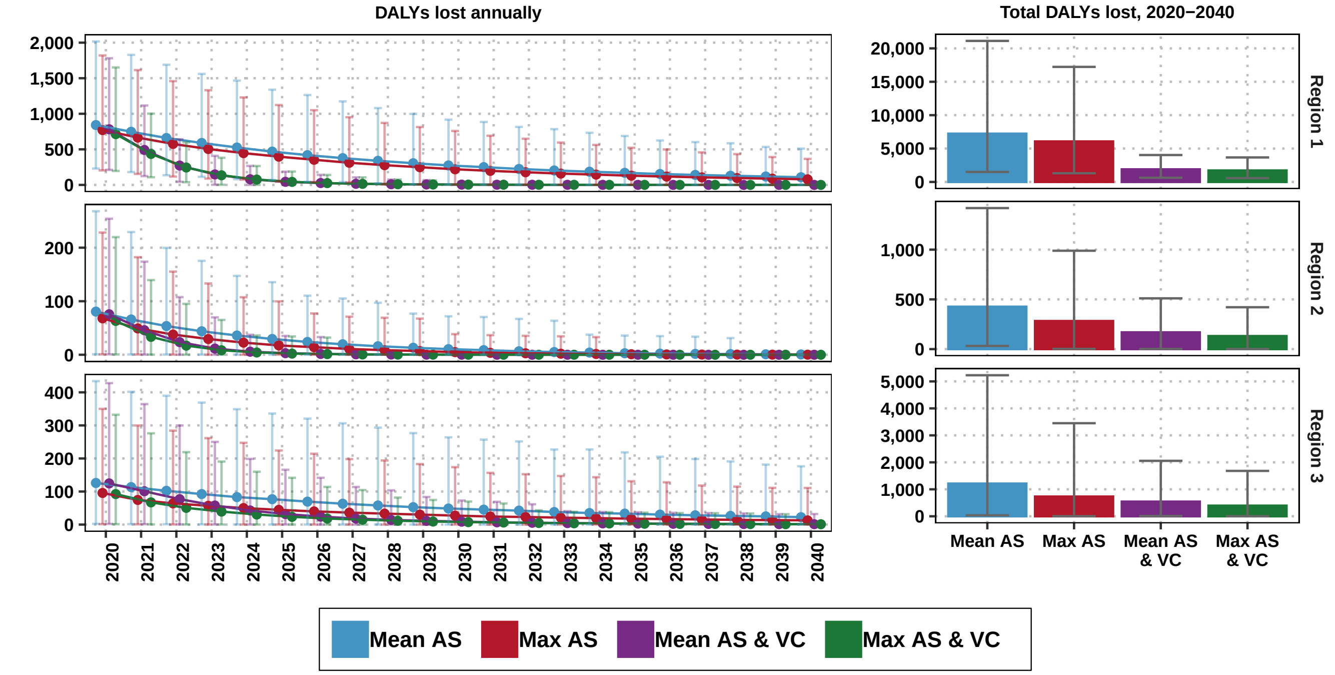

Fig. S3. Components of mean annual and cumulative costs, by strategy and location. Displayed costs are not discounted. Treatment costs, indicated in purple, are shown here although they are so small as to be hardly visible.

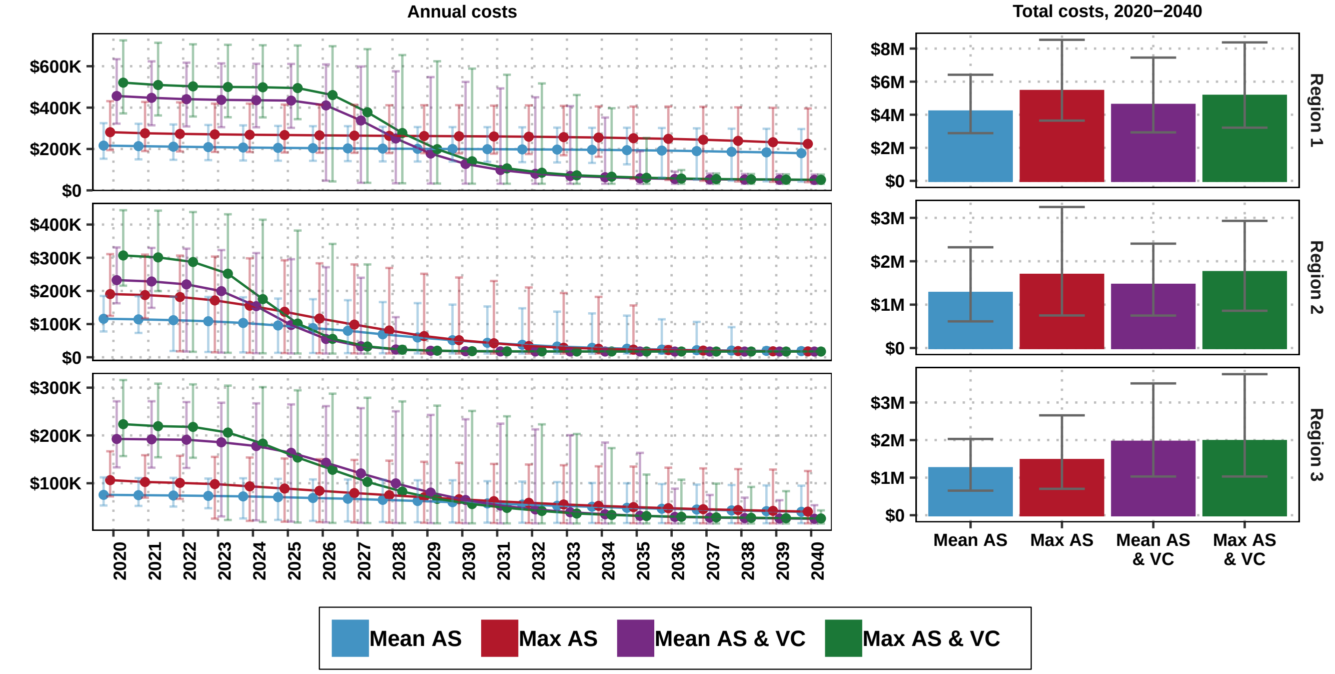

Fig. S4. Disability-adjusted life-years (DALYs) lost per strategy by location, 2020-2040. DALYs are not discounted. Estimates shown are means and their 95% predictive intervals (PI).

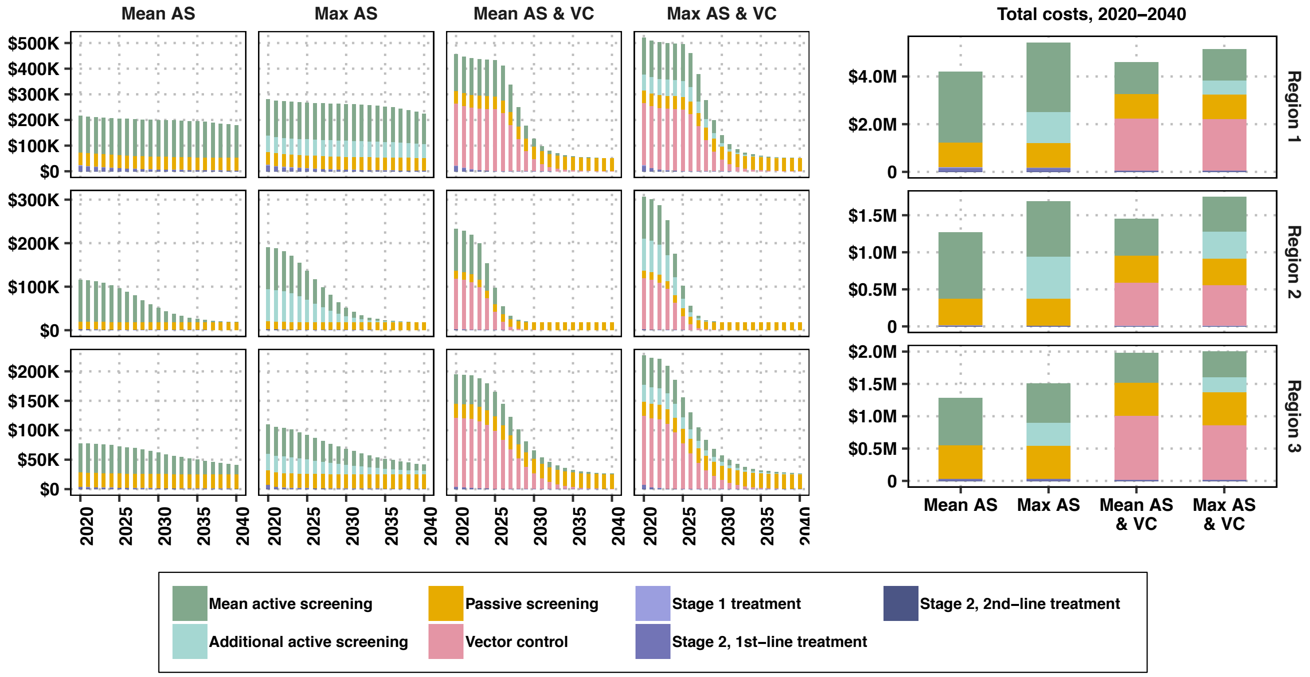

Fig. S5. Costs per strategy by location, 2020-2040. Costs are not discounted. Estimates shown are means and their 95% predictive intervals (PI).

References

1. KS Rock, SJ Torr, C Lumbala, MJ Keeling, Predicting the Impact of Intervention Strategies for Sleeping Sickness in Two High-Endemicity Health Zones of the Democratic Republic of Congo. *PLOS Neglected Trop. Dis.* **11**, 1–17 (2017).
2. RE Crump, et al., Quantifying epidemiological drivers of gambiense human African Trypanosomiasis across the Democratic Republic of Congo. *PLOS Comput. Biol.* **17**, e1008532 (2021).
3. M Antillon, et al., Cost-effectiveness of sleeping sickness elimination campaigns in five settings of the Democratic Republic of Congo (2020).
4. CI Huang, et al., Identifying regions for enhanced control of gambiense sleeping sickness in the Democratic Republic of Congo (2020).
5. F Checchi, F Chappuis, U Karunakara, G Priotto, D Chandramohan, Accuracy of five algorithms to diagnose gambiense human african trypanosomiasis. *PLOS Neglected Trop. Dis.* **5** (2011).
6. PR Bessell, et al., Cost-effectiveness of using a rapid diagnostic test to screen for human African trypanosomiasis in the Democratic Republic of the Congo. *PLOS ONE* **13**, e0204335 (2018).
7. WHO Department of Control of Neglected Tropical Diseases, WHO interim guidelines for the treatment of gambiense human African trypanosomiasis, (World Health Organization, Geneva, Switzerland), Technical report (2019).
8. M Lehane, et al., Tsetse Control and the Elimination of Gambian Sleeping Sickness. *PLOS Neglected Trop. Dis.* **10**, e0004437 (2016).
9. NJ Mbewe, et al., Sticky small target: an effective sampling tool for tsetse fly Glossina fuscipes fuscipes Newstead 1910. *Parasites & Vectors* **11**, 268 (2018).
10. I Tirados, et al., Tsetse Control and Gambian Sleeping Sickness; Implications for Control Strategy. *PLOS Neglected Trop. Dis.* **9**, e0003822 (2015).
11. I Tirados, et al., Impact of tiny targets on Glossina fuscipes quanzensis, the primary vector of human African trypanosomiasis in the Democratic Republic of the Congo. *PLOS Neglected Trop. Dis.* **14**, e0008270 (2020).
12. MH Mahamat, et al., Adding tsetse control to medical activities contributes to decreasing transmission of sleeping sickness in the Mandoul focus (Chad). *PLOS Neglected Trop. Dis.* **11**, 1–19 (2017).
13. M Kagabadouno, et al., Ebola outbreak brings to light an unforeseen impact of tsetse control on sleeping sickness transmission in Guinea. *bioRxiv* (2018).
14. M Aliee, KS Rock, MJ Keeling, Estimating the distribution of time to extinction of infectious diseases in mean-field approaches. *J. The Royal Soc. Interface* **17**, 20200540 (2020).
15. WR Dowdle, The principles of disease elimination and eradication. *Bull. World Heal. Organ.* **76**, 22–25 (1998).
16. WHO Expert Committee on human African trypanosomiasis, Control and surveillance of human African trypanosomiasis: report of a WHO expert committee, Technical report (2013).
17. AA Stinnett, AD Paltiel, Estimating CE Ratios under Second-order Uncertainty. *Med. Decis. Mak.* **17**, 483–489 (1997).
